# Health Financing and Functional Health Expenditure Allocation in Relation to Oral Health: A Longitudinal Ecological Panel Study of EU Member States Between 2016 and 2023

**DOI:** 10.3390/healthcare14213659

**Published:** 2026-10-28

**Authors:** Cassandra Lupita, Anca-Cristina Perpelea, Iulia Muntean, Oana-Ramona Lobonț, Laura-Cristina Rusu

**Affiliations:** 1 Department of Oral Pathology, Multidisciplinary Center for Research, Evaluation, Diagnosis and Therapies in Oral Medicine, “Victor Babes” University of Medicine and Pharmacy Timisoara, 2 Eftimie Murgu Sq., 300041 Timisoara, Romaniaiulia.sauciur@umft.ro (I.M.); laura.rusu@umft.ro (L.-C.R.); 2 Department of Organization, Professional Legislation and Management of the Dental Office, Faculty of Dental Medicine, “Carol Davila” University of Medicine and Pharmacy, 17-23 Plevnei Street, 020021 Bucharest, Romania; 3 Finance, Business Information Systems and Modeling Department, Faculty of Economics and Business Administration, West University of Timisoara, 300115 Timisoara, Romania; oana.lobont@e-uvt.ro

**Keywords:** health expenditure, resource allocation, allocative efficiency, oral health, health policy, European Union

## Abstract

**Highlights:**

**What are the implications of the main findings?**

**1. Implications for policy-makers**

- General preventive healthcare spending is not an indicator of spending on oral disease prevention, as the HC.6 classification includes initiatives covering the whole health sector.
- The specific spending on oral health care must be measured independently by governments and broken down into prevention, early detection, treatment, and rehabilitation.
- Funding for preventive measures must be tied to oral health initiatives, population coverage, service use, and access.
- Assessments of oral health policy must include both expenditures and data about the nature of the initiative, dental services availability, utilization rates, unmet needs, and impacts.
- The European Union institutions must encourage a harmonization of oral health expenditures and service coverage reporting for comparison across member states.

**2. Implications for public**

- This study provides an exploratory assessment of whether changes in system-wide preventive and curative healthcare expenditure are associated with changes in modeled oral-disease prevalence across EU Member States. Because these expenditure categories cover all disease areas, the observed associations should be interpreted as reflecting broader health-system allocation patterns rather than oral-health-specific investment.
- The findings can help identify patterns for further investigation, while their interpretation requires caution regarding the ecological design, the limited within-country variation, and the modeled nature of the outcomes.

**Abstract:**

Background: Health financing decisions allocate resources across preventive and curative functions, but the relationship between system-wide expenditure composition and population oral health is unclear. This study assessed country-level associations between general healthcare expenditure and modeled oral disease prevalence in EU Member States. Methods: We assembled a 2016–2023 ecological panel for 27 Member States, matching Eurostat expenditure data with Global Burden of Disease (GBD) 2023 prevalence estimates for caries of permanent teeth (all ages) and periodontal diseases (age-standardized). Expenditure was examined at per-capita levels, functional shares, and the Preventive Expenditure Share (PES). Two-way fixed-effects models estimated within-country associations, adjusting for country and year effects and, in extended models, GDP per capita and dentist density. The complete panel comprised 216 country-years; primary HC.1 models included 192 observations from 24 countries. Results: In GDP-adjusted models, a 10% higher preventive expenditure level was associated with a 0.028 percentage-point higher caries prevalence (β = 0.291, 95% CI 0.028–0.554; *p* = 0.032); this association was not reproduced in the first-difference model and was specification-sensitive. Curative expenditure was positively associated with periodontal prevalence (β = 0.416, 95% CI 0.038–0.794; *p* = 0.032), equivalent to 0.040 percentage points per 10% expenditure difference, and this association was more consistent across reported sensitivity analyses. PES and functional expenditure shares were not associated with either outcome; the log P/C ratio was positively associated with caries, whereas its untransformed form was not. Conclusions: These ecological estimates describe associations between broad system-wide expenditure categories and modeled oral-health outcomes. They do not identify oral-health-specific spending or establish causal effects, and they cannot show that preventive dentistry is ineffective. Disease-specific expenditure, service delivery, and coverage data are needed to assess oral-health policy performance.

## 1. Introduction

Health systems have become increasingly obliged to manage their healthcare resources in an efficient manner amid growing needs for prevention and treatment services under budget constraints. Financing of health care and priority setting in this area are thus key policy instruments as they define which services will be financed, for which individuals, and how much health value they can produce [1,2,3,4]. The effectiveness of health systems does not only depend on the amount of financial input, but also on governance, provision of services, effective coverage, and quality.

Oral diseases provide a consequential test of this resource-conversion process. They are amongst the most prevalent non-communicable disorders worldwide: in 2019, an estimated 3.5 billion people suffered from them, out of which 2.5 billion people suffered from untreated caries of permanent teeth, and 1 billion people were suffering from advanced periodontal diseases [5,6]. Despite being highly preventable, these diseases are known to predominantly affect people who suffer from poverty, instability, and lack of access to dental care [7,8]. Apart from pain and tooth loss, oral diseases lead to malnutrition, participation, productivity losses, and economic burden [9,10,11].

Countries have to make decisions on not just the amount that they should allocate for healthcare, but also how to allocate and use these funds within prevention, treatment, rehabilitation, and other healthcare services. While increased health spending was often linked to better health outcomes of populations, this association depends on the country’s level of economic development, its governance capacity, institutional efficiency, and the type of outcome measured [12,13,14,15]. This means that total health spending can be an incomplete measure of health system efficiency. Resource allocation consequently represents one of the central policy choices affecting health-system performance.

The importance of expenditure composition is grounded in health capital theory, health production theory, and allocative efficiency. Health is conceptualized as a stock maintained through investment and as an output generated through financial resources, healthcare inputs, socioeconomic conditions, technology, and institutional capacity [16,17]. Allocative efficiency concerns whether available resources fund the combination of services capable of generating the greatest health benefit [18]. While preventive spending may help maintain health capital through the reduction of disease prevalence, early detection, and disease progression, curative spending focuses on existing diseases and needs. Nevertheless, preventive spending does not necessarily imply saving money since the efficacy of this kind of spending relies on many other factors such as program contents, quality, scope, implementation, and coverage [19,20]. The relevant policy question is therefore whether the spending mix balances the avoidance of future disease with the treatment of current needs.

The System of Health Accounts 2011 [21] allows for an international comparison of expenditure by function. The curative care category (HC.1) is defined by the provision of treatment aimed at alleviating symptoms, mitigating the severity of illness, or restoring good health, while the preventive care (HC.6) category consists of immunization, early detection, health surveillance, risk information, and counseling activities [21]. These categories describe different health system functions rather than disease-specific expenditure. In 2022, preventive care amounted to 5.5% of total current healthcare expenditure in the European Union (EU), although there were large variations across Member States [22]. The structure of this category of expenditure was also modified due to the COVID-19 pandemic because of its inclusion of expenditure related to testing, vaccination, surveillance, and coordination [23,24].

Although previous studies have examined total healthcare expenditure, health-system efficiency, dental care access, insurance coverage, and the cost-effectiveness of preventive dental interventions, evidence on expenditure composition remains limited. Research on preventive-curative allocation has focused primarily on economic development or broad population outcomes rather than oral health [25]. Priority-setting research further shows that high performance depends on transparent structures, processes, evidence use, implementation, and outcomes rather than allocation decisions alone [2]. To our knowledge, no longitudinal cross-country study has evaluated whether the allocation of general healthcare expenditure between preventive and curative functions is associated with population-level oral health outcomes. This is a specific policy gap because expenditure composition is directly amenable to government action and may influence performance independently of overall spending, whereas disease burden itself is not directly modifiable through accounting choices.

The study of oral health is especially useful for addressing the identified knowledge gap. Caries and periodontal diseases are progressive illnesses where health promotion, risk reduction, early detection, and interventions can prevent their occurrence and development [7,26,27]. Oral healthcare services are often excluded from medical insurance, and there are considerable differences between European countries in terms of dental entitlements, benefit packages, workforce organization, cost sharing, service utilization, and unmet need [28,29,30,31].

This difference gives rise to a significant measurement problem. The HC.6 spending is not dedicated to dentistry, but to vaccination, screening, surveillance, occupational health care, risk communication, and emergency preparedness; HC.1 involves curative services for all diseases. Thus, these classifications need to be understood as measures of health system spending structure, not specific oral health expenditure and performance. Nevertheless, oral health serves as a sensitive case for analysis to study how system-wide resource allocation is associated with outcomes. If the relation between general functional spending patterns and oral health is weak, disease-specific resource allocation, implementation, service coverage, access, quality, and use will provide relevant data. As the distribution of resources into preventive and curative functions can be influenced by governments, such research can help to make informed decisions about priority setting, financing and effective use of scarce public resources.

The EU is a proper setting because it consists of Member States that have the same accounting classification of expenditures but differ in their economic performance, health systems structure, prevention strategy, dental coverage, workforce and disease burden [23,30]. Country-level longitudinal data allow assessing changes within countries, controlling for national stable factors, and accounting for common shocks.

Accordingly, this study evaluated whether within-country changes in broad, system-wide healthcare expenditure categories were associated with changes in modeled population-level oral health across EU Member States between 2016 and 2023. Preventive and curative expenditure were examined as absolute levels, functional shares, and through the Preventive Expenditure Share (PES), a descriptive measure of the preventive share within the combined preventive and curative expenditure envelope. The outcomes were GBD prevalence estimates for caries of permanent teeth (all ages) and periodontal diseases (age-standardized). The analysis accounted for economic capacity, dentist density in extended models, country fixed effects, and common year effects. The design cannot identify expenditure specifically directed to oral health or estimate causal effects.

This study contributes by providing, to our knowledge, the first longitudinal cross-country analysis of functional healthcare expenditure allocation and population oral health; using PES as a descriptive measure of expenditure composition rather than an index of health-system orientation, efficiency, or performance; comparing absolute expenditure with relative allocation and functional shares; and integrating health economics, oral health research, and policy analysis. Determining whether allocation is associated with oral health is relevant for governments seeking to strengthen health-system performance under constrained budgets. If no association is identified, broad expenditure categories may be insufficient for performance assessment without information on program content, disease-specific investment, implementation, effective coverage, service quality, and uptake. Based on health capital theory, health production theory, and allocative efficiency, the following hypotheses were evaluated:

**H1.**  *Higher preventive healthcare expenditure is associated with lower population-level oral disease prevalence across EU Member States.*

**H2.**  *After accounting for national economic capacity and healthcare-system characteristics, higher curative healthcare expenditure is not associated with lower population-level oral disease prevalence.*

**H3.**  *A greater preventive share within combined preventive and treatment-oriented expenditure, as summarized by the PES, is associated with lower population-level oral disease prevalence.*

## 2. Materials and Methods

### 2.1. Study Design and Policy Framework

This longitudinal ecological panel study investigated country-level associations between system-wide functional healthcare expenditure and modeled population oral-health outcomes across EU Member States from 2016 to 2023. The country-year was the unit of analysis. The period was the longest interval for which the selected Eurostat expenditure data and GBD 2023 oral-health estimates were jointly available for the study. It covered pre-pandemic, pandemic, and early post-pandemic years. The estimand is a within-country association conditional on country and year fixed effects, not an individual-level relationship or a causal effect. Annual trends in the country-level means and one-year lagged expenditure associations were examined as supplementary analyses; the short lag is not interpreted as representing the biological latency of cumulative oral diseases.

**Figure 1:**
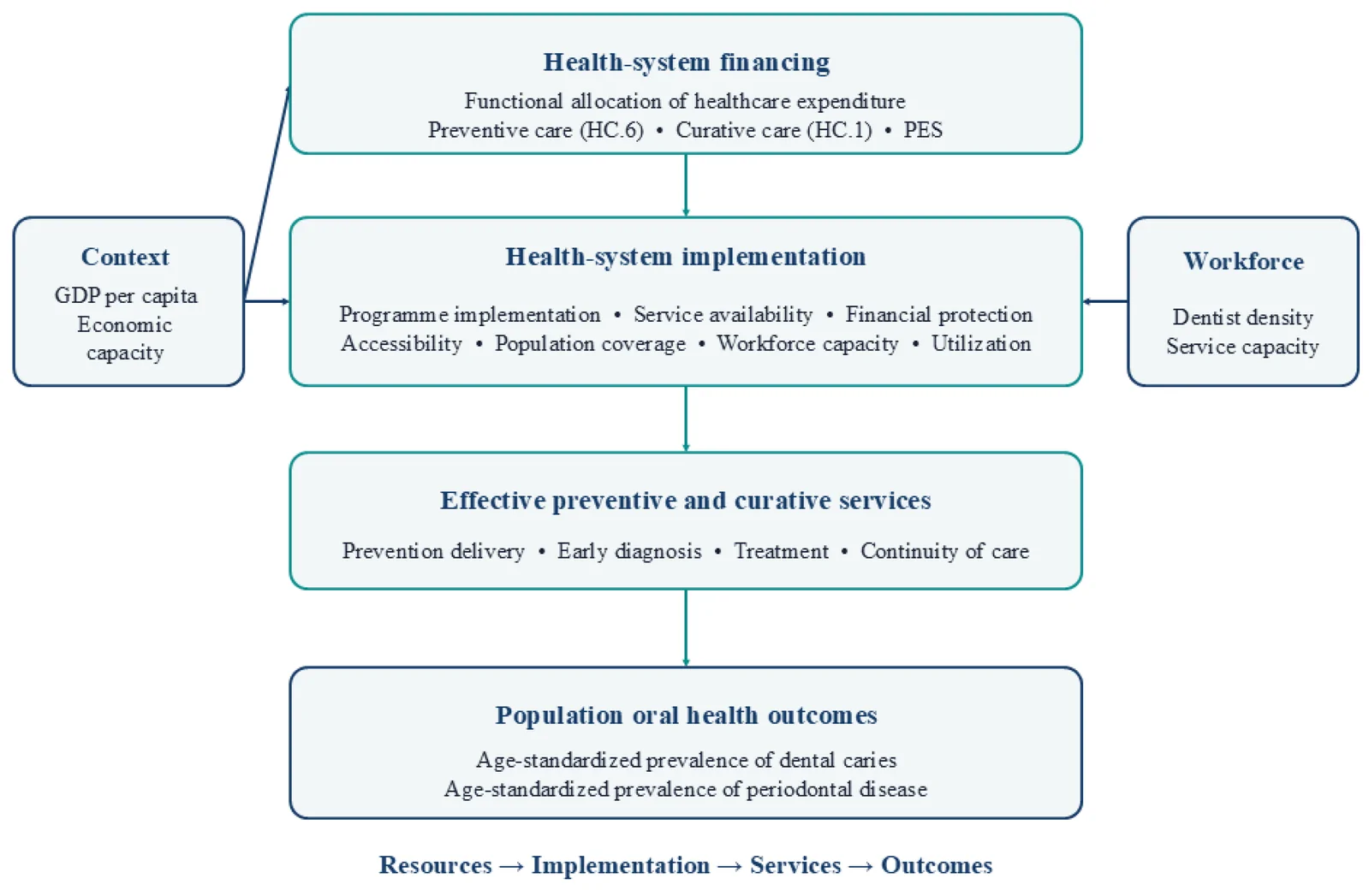
Conceptual pathway hypothesizing links between health-system financing, functional expenditure allocation, and population oral-health outcomes; the arrows represent proposed relationships, not estimated causal effects. HC.1, curative care; HC.6, preventive care; GDP, gross domestic product; PES, Preventive Expenditure Share.

Expenditure allocation was assessed from three complementary perspectives: absolute preventive and curative expenditure; the preventive share within selected preventive and treatment-oriented functions, summarized by the Preventive Expenditure Share (PES); and functional shares of total current healthcare expenditure. The outcomes were prevalence estimates for caries of permanent teeth (all ages) and periodontal diseases (age-standardized), analyzed separately. Expenditure functions conformed to the System of Health Accounts 2011 classification [21]. Figure 1 illustrates the analytical framework and hypothesized pathways linking healthcare expenditure allocation, contextual factors, service conversion, and oral health outcomes.

Because the analysis used aggregated observational data, coefficients represent ecological associations at the health-system level. They should not be interpreted as causal effects or as evidence of individual-level relationships.

### 2.2. Data Sources and Panel Construction

The panel combined country-level information from IHME and Eurostat. Oral-health outcomes were extracted from the Global Burden of Disease (GBD) 2023 results tool [32] for each EU Member State and year 2016–2023, using measure = prevalence, metric = Percent, and sex = both. The extracted cause labels were “Caries of permanent teeth” and “Periodontal diseases,” as defined in the GBD cause hierarchy. The age setting was “All ages” for caries and “Age-standardized” for periodontal disease; the outcomes are therefore not age-standardized on a common basis, and their coefficient magnitudes should not be directly compared. Both outcomes are modeled estimates, not repeated national clinical measurements. Downloaded values were returned as proportions despite the “Percent” metric label; values were multiplied by 100, checked against source estimates, and analyzed as percentages. Point estimates were used in regressions. Lower and upper 95% uncertainty limits were retained for descriptive assessment and an exploratory inverse-variance-weighted sensitivity analysis, but GBD model uncertainty was not fully propagated through the primary regressions.

Healthcare expenditure was obtained from the Eurostat Health care expenditure by function dataset (hlth_sha11_hc). Selected functions were total current healthcare expenditure, curative care (HC.1), combined curative and rehabilitative care (HC.1 + HC.2), and preventive care (HC.6). The primary analysis used purchasing power standards (PPS) per inhabitant to account for differences in national price levels. Eurostat-reported functional shares were retained for sensitivity analyses.

Data on GDP per capita were sourced from the Eurostat GDP and main components per capita dataset (nama_10_pc) in current PPS per capita. Data on dental workforce density were sourced from the Eurostat Healthcare personnel statistics dataset (hlth_rs_prs2) in dentists per 100,000 inhabitants.

Databases were restructured into country-year format for 2016–2023, matched by country identifier, and merged one-to-one. Eurostat status flags were retained in the data audit and checked against dataset metadata; observations marked unavailable (“:”) were treated as missing, while numeric values carrying provisional, break-in-series, or estimated flags were retained with their flags. Expenditure values were not imputed. Potentially extreme values were checked against the source extracts; no automatic winsorization or outlier deletion was applied. Models used complete observations for their respective variables.

The master panel comprised 27 EU Member States and 216 country-year observations. Exact HC.1 expenditure was unavailable for Ireland, Italy, and Sweden in all eight years; therefore, primary HC.1 models used a balanced panel of 24 countries and 192 observations. The alternative HC.1 + HC.2 analysis retained all 27 Member States. These samples represent different country sets, so complete-panel estimates are not direct replications on an identical population. Dentist-density data were available for 209 of 216 country-years; the seven missing observations were Luxembourg in 2018–2023 and Sweden in 2023. Because Sweden was already excluded from the HC.1 panel, workforce-adjusted HC.1 models contained 186 observations, with all six additional missing observations from Luxembourg. Missing values were not imputed (Table S4).

### 2.3. Variables and Measurement

The outcomes were GBD modeled prevalence estimates for caries of permanent teeth (all ages) and periodontal diseases (age-standardized), expressed as percentages; regression coefficients therefore represent percentage-point differences in prevalence. They are not annual standardized clinical examination results. IHME point estimates were used in the primary regressions. The lower and upper GBD 95% uncertainty limits were retained for descriptive assessment and an exploratory weighted sensitivity analysis but were not propagated as outcome uncertainty in the primary regressions.

The specification was kept parsimonious to focus on broad functional expenditure associations. Harmonized annual covariates were unavailable for several potentially important determinants, including dental coverage, out-of-pocket costs, service use, smoking, fluoride exposure, and oral-health policies. Country fixed effects account for stable country characteristics, but not for unmeasured time-varying confounding. The estimates remain observational and may also reflect reverse allocation from disease burden to expenditure.

The main exposures were preventive care expenditure (HC.6) and curative care expenditure (HC.1), measured in PPS per inhabitant. Natural-log transformation reduced right skewness; the resulting coefficients describe percentage-point differences in prevalence associated with proportional differences in expenditure. Curative plus rehabilitative expenditure (HC.1 + HC.2) served as the treatment-oriented alternative for the complete EU-27 panel.

The Preventive Expenditure Share (PES) was constructed as a descriptive indicator of the relative share of preventive expenditure within the combined preventive and curative expenditure envelope:

PES_it_ = [P_it_/(P_it_ + C_it_)] × 100 (1)

where P_it_ and C_it_ denote preventive and curative expenditure, respectively, for country *i* in year *t*. Higher PES values indicate that a larger proportion of expenditure is allocated to prevention relative to treatment. PES describes expenditure composition and should not be interpreted as a measure of allocative efficiency, preventive policy effectiveness, health-system performance, or strategic priority. It was modeled separately from its components. An alternative specification replaced HC.1 with HC.1 + HC.2 for the complete EU-27 panel. Because other functions were excluded from the denominator, PES is not the preventive share of total healthcare expenditure.

The ratio of preventive to curative expenditure was examined in two separate sensitivity specifications. GDP per capita was included as an indicator of economic capacity. Dentist density was used as a measure of workforce availability, not as a direct measure of access, affordability, coverage, or service use. Eurostat provided practicing dentists for 174 country-years, professionally active dentists for 8, and licensed dentists for 27 of the 209 observations with a workforce value. The source definition was retained for each country-year rather than treating these categories as interchangeable; this heterogeneity limits workforce-adjusted estimates.

### 2.4. Econometric Analysis

Variables were checked for integrity, distribution, consistency through time, and the potential influence of observations. Continuous variables were described through their means, standard deviation, median, interquartile range, minimum, and maximum. Also analyzed were country and year trends and correlations among pairs of variables.

The analytical approach estimated within-country associations between changes in expenditure allocation and changes in modeled oral-health outcomes, controlling for time-invariant country characteristics and common year shocks. Fixed effects were selected because institutional, financing, governance, and organizational features may correlate with expenditure allocation and are relatively stable. Within-country variation over only eight years was limited for some outcomes and exposures; accordingly, estimates may be imprecise and sensitive to model specification. The analysis included the available EU Member States rather than a sample drawn from a larger population; no prospective power calculation was performed, and the study cannot be assumed to have adequate power for small associations or secondary analyses.

Separate two-way fixed-effects regressions were estimated for caries and periodontal disease:

Y_it_ = β_0_ + β_1_ ln(P_it_) + β_2_ ln(C_it_) + γ′X_it_ + α_i_ + λ_t_ + ε_it_ (2)

where Y_it_ represented the oral health outcome in country *i* and year *t*; P_it_ and C_it_ represented preventive and curative expenditure, respectively; X_it_ is a vector of time-varying control variables; γ is the corresponding coefficient vector; α_i_ represents country fixed effects; λ_t_ represents year fixed effects; and ε_it_ is the idiosyncratic error term.

The country fixed effects allowed controlling for any country-fixed attributes that are both observed and unobserved, whereas the year fixed effects accounted for any common shocks, such as economic developments, the pandemic, interruptions in the use of health services, and changes in the reporting system.

Model 1 included preventive and curative expenditure; Model 2 additionally included GDP per capita; Model 3 additionally included dentist density. PES and functional expenditure shares were analyzed separately. As robustness specifications, P/C and its natural logarithm were estimated in GDP-adjusted two-way fixed-effects models. Because outcomes are measured in percentage points and expenditure is log-transformed, expenditure coefficients are semi-elasticities: β × ln(1.10) gives the estimated percentage-point difference associated with a 10% expenditure difference. Preventive-versus-curative coefficients were compared using Wald tests.

Standard errors were clustered by country to allow heteroskedasticity and serial correlation within countries. With 24 country clusters in the primary models (and 27 in complete-panel models), conventional cluster-robust standard errors and associated confidence intervals and *p*-values may be unreliable in finite samples; results should therefore be interpreted cautiously, particularly for marginal *p*-values and sensitivity models. Results are reported with 95% confidence intervals and two-sided *p*-values. Driscoll–Kraay inference was also examined as a sensitivity check, but it does not remove the small-cluster limitation and was interpreted cautiously because there are only eight annual periods.

### 2.5. Diagnostic and Sensitivity Analyses

Multicollinearity was assessed using correlations and variance inflation factors. Panel diagnostics evaluated serial correlation, groupwise heteroskedasticity, and cross-sectional dependence. Driscoll–Kraay standard errors were calculated when cross-sectional dependence was detected but interpreted cautiously because the panel contained only eight annual periods. The primary analyses were the two-way fixed-effects models of log HC.6 and log HC.1, with Model 2 (GDP-adjusted) designated as the principal adjusted specification. Other outcomes, functional shares, ratios, and estimator variants were treated as secondary or sensitivity analyses. Given the number of related models and outcomes, isolated nominal *p* < 0.05 results are interpreted as exploratory; no multiplicity-adjusted inferential claims are made.

Robustness was evaluated by:

- replacing HC.1 with HC.1 + HC.2 in the complete EU-27 panel;
- replacing expenditure levels with functional expenditure shares;
- evaluating the preventive-to-curative expenditure ratio (P/C) in levels and natural-log form;
- lagging explanatory variables by one year;
- estimating first-difference models;
- excluding 2020–2021;
- introducing dental workforce density in extended and balanced-sample models;
- conducting leave-one-country-out analyses;
- comparing fixed-effects estimates with pooled, random-effects, and alternative-error specifications.

Lagged models were included as a temporal sensitivity analysis, not as a solution to simultaneity or reverse causality. The one-year lag was selected as a feasible short-panel specification that preserves seven annual transitions; it is not assumed to represent bio-logically sufficient latency for caries or periodontal disease, which accumulate over longer periods. In the lagged fixed-effects model, the preventive-expenditure estimate for caries was positive (β = 0.282; 95% CI 0.026 to 0.538; *p* = 0.032), while the first-difference estimate was not statistically significant (β = 0.033; 95% CI −0.103 to 0.170; *p* = 0.617). This contrast indicates specification sensitivity, not a resolved temporal effect. First-difference models were supplementary because differencing may amplify measurement error when annual changes in modeled prevalence are small. Residual endogeneity and reverse allocation from disease burden to expenditure cannot be excluded. As an exploratory check, inverse-variance weights were derived from each GBD 95% uncertainty-interval width, treating width/3.92 as an approximate standard error; these weighted estimates do not capture all GBD model uncertainty (Table S3).

Preprocessing, paneling, analysis, and visualization were carried out in Python 3.14 using pandas, NumPy, SciPy, and Matplotlib. Descriptive statistics and correlations were verified independently in JASP 0.96.0. All transformations were done in scripts that operated on copies of the raw data.

### 2.6. Ethical Issues/Statement

This study used aggregated country-level data from international statistical databases and did not involve individual participants, patient records, biological samples, interventions, or personally identifiable information. Institutional ethical approval and informed consent were therefore not required. All data were analyzed and reported at the national level.

## 3. Results

### 3.1. Panel Characteristics and Expenditure Patterns

The complete panel comprised 216 country-year observations from 27 EU Member States between 2016 and 2023. The primary sample based on exact curative-care expenditure (HC.1) included 192 observations from 24 countries, while analyses using combined curative and rehabilitative-care expenditure (HC.1 + HC.2) retained the complete EU-27 panel.

Mean prevalence was 34.80% (SD = 6.00) for caries of permanent teeth (all ages) and 11.20% (SD = 4.02) for age-standardized periodontal diseases. Mean preventive expenditure was 97.05 PPS per inhabitant, compared with 1373.30 PPS for curative expenditure and 1517.40 PPS for combined curative and rehabilitative expenditure. The HC.1-based PES averaged 5.74%, while preventive and curative care represented 3.28% and 52.34% of total current healthcare expenditure, respectively (Table 1). Descriptive statistics showed substantial between-country variation in expenditure allocation despite comparatively modest variation in modeled oral-disease prevalence, supporting cautious analysis of within-country change.

Caries prevalence declined from an annual cross-country mean of 35.01% in 2016 to 34.42% in 2023, while periodontal disease prevalence changed from 11.17% to 11.07%. Preventive expenditure increased from 68.34 PPS per inhabitant in 2019 to 180.04 PPS in 2021 before declining to 104.28 PPS in 2023. The 2020–2021 peak coincided with the COVID-19 pandemic and the temporary expansion of prevention-related activities across health systems; it did not represent an oral-health-specific trend. Curative expenditure increased more gradually, from 1148.85 PPS in 2016 to 1657.05 PPS in 2023. Annual trends and pooled correlations are reported in Table S1.

### 3.2. Preventive and Curative Expenditure and Oral Health Outcomes

Table 2 presents the two-way fixed-effects estimates. Model 1 included preventive and curative expenditure, Model 2 added GDP per capita, and Model 3 added dental workforce density. All models included country and year fixed effects.

#### 3.2.1. Caries Prevalence

In the GDP-adjusted model, preventive expenditure was positively associated with modeled caries prevalence (β = 0.291 percentage points per log-unit; 95% CI 0.028 to 0.554; *p* = 0.032). A 10% expenditure difference corresponds to an estimated 0.028 percentage-point difference (β × ln(1.10)). This association was specification-sensitive: Model 1 estimated β = 0.299 (95% CI −0.015 to 0.613; *p* = 0.061), while Model 3 estimated β = 0.237 (95% CI 0.044 to 0.431; *p* = 0.018). The one-year lagged estimate was also positive (β = 0.282; 95% CI 0.026 to 0.538; *p* = 0.032), but the first-difference estimate was near zero and non-significant (β = 0.033; 95% CI −0.103 to 0.170; *p* = 0.617). Excluding 2020–2021 yielded β = 0.410 (95% CI 0.108 to 0.711; *p* = 0.010). Thus, the direction was generally positive across level-based specifications, but the estimate was not supported by the first-difference analysis and does not establish a temporal or causal association. Given the observational design and broad HC.6 definition, this is not evidence that preventive expenditure increases caries prevalence. Curative expenditure was not associated with caries after GDP adjustment (β = −0.107; 95% CI −0.798 to 0.584; *p* = 0.751).

After additional adjustment for dentist density, the preventive-expenditure coefficient remained positive (β = 0.237; 95% CI 0.044 to 0.431; *p* = 0.018), while curative expenditure remained non-significant. Dentist density had an inverse but imprecisely estimated coefficient (β = −0.0068; 95% CI −0.0146 to 0.0011; *p* = 0.088). These estimates are conditional country-level associations, not effects of individual expenditure changes.

#### 3.2.2. Periodontal Disease Prevalence

Curative expenditure was positively associated with periodontal disease prevalence after adjustment for GDP per capita (β = 0.416; 95% CI: 0.038 to 0.794; *p* = 0.032), corresponding to an estimated increase of 0.040 percentage points for a 10% increase in expenditure. The association remained after adjustment for dental workforce density (β = 0.388; 95% CI: 0.027 to 0.749; *p* = 0.036).

Preventive expenditure and GDP per capita were not independently associated with periodontal disease prevalence. Dental workforce density was inversely associated with prevalence in Model 3 (β = −0.0075; 95% CI: −0.0128 to −0.0022; *p* = 0.008), corresponding to an estimated reduction of 0.075 percentage points for 10 additional dentists per 100 000 inhabitants. The curative coefficient was significantly larger than the preventive coefficient in Models 2 (Wald *p* = 0.016) and 3 (Wald *p* = 0.026).

#### 3.2.3. Preventive Expenditure Share, P/C Ratio, and Functional Expenditure Shares

The relative-allocation results depended on the functional form. For caries, PES and the untransformed P/C ratio were not statistically associated with prevalence (PES β = 0.023, 95% CI −0.013 to 0.058; *p* = 0.196; P/C β = 1.570, 95% CI −1.136 to 4.275; *p* = 0.242), whereas ln(P/C) was positively associated (β = 0.281, 95% CI 0.026 to 0.537; *p* = 0.032). The complete-panel ln[P/(C + R)] estimate was also positive (β = 0.274, 95% CI 0.022 to 0.527; *p* = 0.034), while the corresponding PES and untransformed ratio estimates were not significant. Because PES is a nonlinear transformation of P/C and the models impose different functional forms, these estimates are not interchangeable; the log-ratio result should be treated as a specification-sensitive secondary finding, not evidence of a general effect of preventive allocation.

For periodontal disease, the HC.1-based PES (β = 0.005; 95% CI −0.020 to 0.031; *p* = 0.666) and its HC.1 + HC.2 alternative (β = 0.035; 95% CI −0.028 to 0.097; *p* = 0.263) were not associated with prevalence. Functional expenditure shares were also non-significant. Neither untransformed P/C (β = 0.383; 95% CI −1.517 to 2.283; *p* = 0.680) nor ln(P/C) (β = 0.052; 95% CI −0.190 to 0.294; *p* = 0.662) was associated with periodontal prevalence (Table 3).

### 3.3. Diagnostic and Sensitivity Analyses

Variance inflation factors ranged from 2.41 to 4.45. Diagnostics identified within-country serial correlation and heteroskedasticity. Cross-sectional dependence was not detected in primary HC.1 models but was observed for periodontal disease in the complete HC.1 + HC.2 panel; supplementary Driscoll–Kraay estimation preserved coefficient directions (Table S2). Replacing HC.1 with HC.1 + HC.2 produced positive associations for preventive expenditure and caries (β = 0.276; 95% CI 0.018 to 0.533; *p* = 0.037) and treatment-oriented expenditure and periodontal prevalence (β = 0.564; 95% CI 0.006 to 1.123; *p* = 0.048). Excluding 2020–2021 yielded a positive preventive-caries coefficient (β = 0.410; 95% CI 0.108 to 0.711; *p* = 0.010) and a positive curative-periodontal coefficient (β = 0.332; 95% CI 0.005 to 0.659; *p* = 0.047). The full estimates are reported in Table S3.

**Figure 2:**
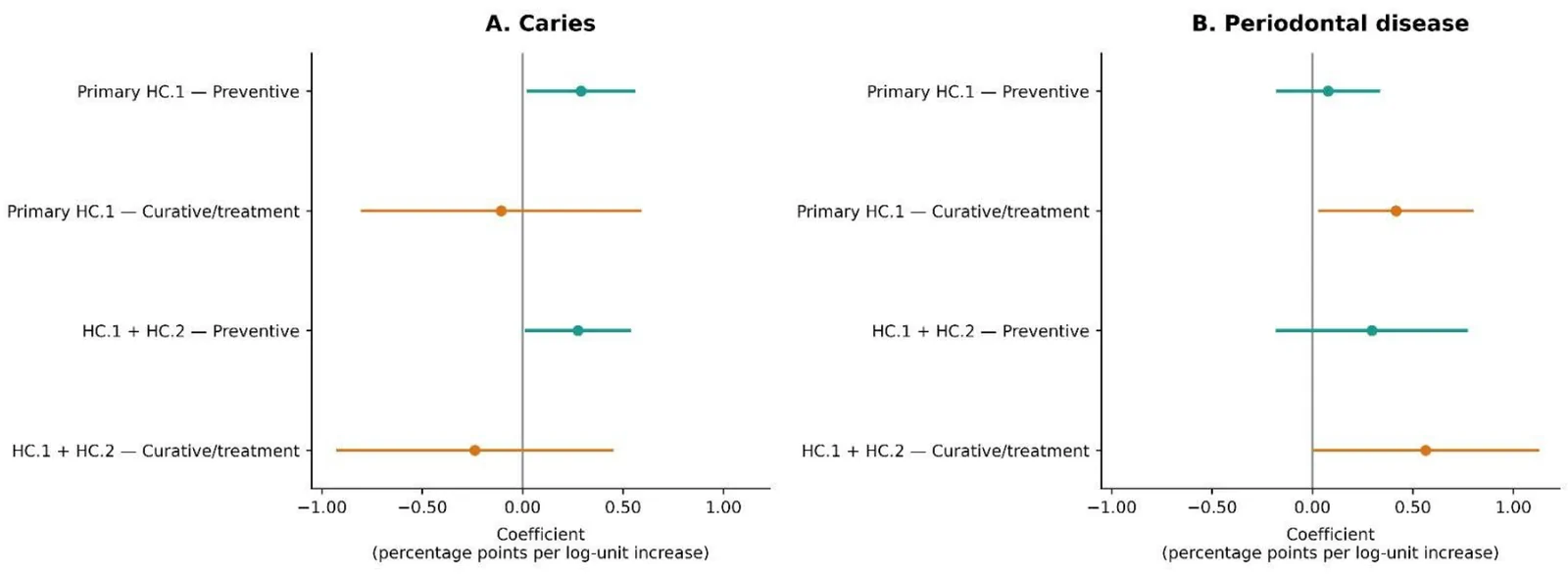
GDP-adjusted fixed-effects esti-mates for preventive and treatment-oriented expenditure in the primary HC.1 and complete HC.1 + HC.2 panel spec-ifications: (**A**) caries prevalence; (**B**) periodontal disease prevalence. Points represent regression coefficients, and horizontal lines indicate 95% confidence intervals based on country-clustered standard errors. All models included GDP per capita, country fixed effects, and year fixed effects. Coefficients are expressed as percentage-point differ-ences in prevalence per log-unit increase in expenditure. HC.1 denotes curative care; HC.2 denotes rehabilitative care.

In the one-year lagged model, the preventive-expenditure/caries association remained positive (β = 0.282; 95% CI 0.026 to 0.538; *p* = 0.032), but the first-difference estimate was not significant (β = 0.033; 95% CI −0.103 to 0.170; *p* = 0.617). The curative-expenditure/periodontal association was positive but less precise in the lagged model (β = 0.418; 95% CI −0.021 to 0.858; *p* = 0.061), whereas it remained positive in first differences (β = 0.262; 95% CI 0.034 to 0.490; *p* = 0.026). The corresponding estimates used 168 observations from 24 countries over seven years. The preventive-caries association also remained positive in the random-effects model (β = 0.284; 95% CI 0.036 to 0.531; *p* = 0.026) and in the workforce-balanced model excluding Luxembourg (β = 0.238; 95% CI 0.044 to 0.432; *p* = 0.019; N = 184; 23 countries). Leave-one-country-out analyses showed no single-country dependence in the sign of the main coefficients (Table S3) (Figure 2).

Overall, sensitivity analyses indicate that the positive preventive-expenditure/caries association is present in level-based and lagged specifications but not in first differences; it should therefore be described as specification-sensitive. The exploratory inverse-variance-weighted model also produced a positive preventive-caries coefficient (β = 0.356; 95% CI 0.021 to 0.691; *p* = 0.038), but the approximate weighting and multiple analyses warrant caution. The curative-expenditure/periodontal association was more consistent, including in first differences, although its lagged estimate was less precise. In the same weighted analysis, the curative-periodontal coefficient was positive (β = 0.421; 95% CI 0.050 to 0.792; *p* = 0.028). These results remain ecological associations and do not distinguish temporal ordering from reverse allocation or time-varying confounding. Relative-allocation findings were form-dependent: the log-ratio was positively associated with caries, whereas PES and the untransformed ratio were not. H1 and H3 were not supported by the primary analyses. H2 was consistent with the absence of a lower prevalence estimate for some outcomes, but its results do not establish whether greater treatment-oriented spending reduces disease burden.

## 4. Discussion

### 4.1. Principal Findings

In this ecological panel, we found no evidence that greater preventive allocation within broad, system-wide expenditure categories was associated with lower modeled caries or periodontal prevalence across EU Member States. The primary adjusted estimate for preventive expenditure and caries was positive but specification-sensitive and was not reproduced in first differences. The positive association between general curative expenditure and modeled periodontal prevalence was more consistent across the reported specifications. These are country-level associations and do not establish causal effects or identify oral-health-specific expenditure.

These findings do not imply that preventive dentistry is ineffective. HC.6 does not identify which oral-health preventive services were delivered, to whom, at what quality, or with what effective coverage. The results address associations between broad system-wide expenditure categories and modeled population outcomes; they cannot test the effectiveness of specific dental prevention programs. Functional expenditure composition alone is therefore an incomplete proxy for health-system performance [1,3,4].

### 4.2. Interpretation and Comparison with Existing Literature

The absence of an inverse association between broad preventive expenditure measures and modeled oral-disease prevalence should not be interpreted as evidence that prevention is ineffective. General healthcare expenditure may not be converted into improved population outcomes uniformly; the relationship can depend on institutional capacity, economic development, governance, service delivery, and how resources are translated into care [12,13,14,15]. Expenditure composition describes functions in national accounts, not necessarily the services delivered or outcomes achieved.

This distinction is particularly important for HC.6, which includes immunization, screening, surveillance, risk communication, occupational health, and emergency preparedness across the entire health system. Only an unidentifiable proportion is directed toward oral health. Increased HC.6 expenditure may therefore occur without improving access to fluoride interventions, preventive dental examinations, periodontal risk management, oral health education, or early treatment.

The COVID-19 period illustrates this issue of measurement. Preventive expenditure increased substantially because of testing, vaccination, surveillance, communication, and emergency-response activities rather than a sustained expansion of conventional prevention [23,24]. Oral disease prevalence changed comparatively little. Although excluding 2020–2021 preserved the principal coefficient patterns, aggregate preventive expenditure cannot be interpreted as equivalent to preventive oral healthcare.

The positive association between broad preventive expenditure and modeled caries prevalence should not be interpreted as evidence that preventive spending increases disease prevalence. Reverse allocation is plausible: countries with greater health needs may increase expenditure in response. The association remained positive in the one-year lagged model, but was not reproduced in first differences, which do not resolve temporal ordering or causality. Longitudinal evidence documents progression of caries in permanent dentition among children and adolescents and cumulative periodontal attachment loss over decades in adult men; these cohorts illustrate why short lags may not capture the course of cumulative oral disease, but they do not establish the appropriate latency for the GBD EU outcomes analyzed here [33,34]. This study did not test long-term latency. The estimates should therefore be read as short-panel associations, not as an assessment of long-term preventive impact.

Preventive dental interventions targeted at specific diseases can reduce the risk of disease development, support early diagnosis, and limit progression [7,26,27,35]. Their effectiveness depends on program design and implementation [19,20]. These findings should therefore be interpreted in light of the broad functional classification of healthcare expenditure.

The positive country-level association between general curative expenditure and modeled periodontal prevalence was more consistent across the reported analyses. It does not indicate that treatment causes periodontal disease. Need-driven allocation is one plausible hypothesis: countries with greater chronic disease burden may devote more resources to treatment. The design cannot distinguish this mechanism from time-varying confounding, changes in ascertainment, or other explanations. Periodontal disease develops and persists over long periods, so a one-year exposure lag in an eight-year panel is unlikely to capture its full latency.

A treatment-oriented system may also spend substantial resources managing established disease without addressing its upstream determinants. Curative expenditure may consequently reflect accumulated need and delayed intervention rather than an effective mechanism for reducing prevalence. Because HC.1 covers all disease areas, however, this interpretation cannot be attributed specifically to periodontal treatment.

The PES results were not statistically significant, but this does not exhaust the relative-allocation evidence. The positive caries association for ln(P/C), alongside non-significant estimates for PES and untransformed P/C, indicates sensitivity to the functional form. As PES is a nonlinear transformation of P/C and the specifications differ, these findings should be reported separately and treated as exploratory. PES remains a descriptive measure of composition, not an indicator of preventive policy effectiveness or health-system performance.

Contextual findings reinforce that workforce density and economic capacity are incomplete descriptors of health-system context. Dentist density was associated with lower periodontal prevalence in the fully adjusted model, but workforce density alone does not establish access, affordability, coverage, quality, or use. Dental benefit packages, financial protection, geographic distribution, and actual utilization remain relevant omitted factors [28,29,30,31].

Research on dental coverage, access, and utilization provides a complementary perspective. These studies address arrangements more directly related to the delivery and use of oral healthcare than the system-wide expenditure categories examined here. Our analysis cannot determine whether changes in HC.6 or HC.1 expenditure altered dental entitlements, financial protection, or service utilization. The findings therefore highlight the need to connect expenditure composition with these intermediate dimensions when evaluating its relationship with oral-health outcomes.

### 4.3. Health Policy Implications

Governments should not assume that increasing expenditure classified as preventive, or mechanically reallocating resources from treatment to prevention, will by itself improve oral health. Under constrained public budgets, priority setting should focus on interventions with demonstrated effectiveness, measurable coverage, implementation capacity, and equitable access rather than on expenditure proportions alone [2]. Functional allocation describes broad policy orientation but cannot determine whether resources reach effective oral health programs.

Expenditure indicators should therefore be combined with measures of oral-health-specific investment, service delivery, effective coverage, dental insurance and financial protection, cost sharing, workforce distribution, utilization, unmet need, quality, equity, and program effectiveness. This supports a shift from expenditure-based monitoring toward performance-oriented health financing, in which allocation is evaluated according to the value generated for population health rather than accounting categories alone [1,3,4,36,37].

Finally, oral health should become an integral part of general health policy. Since there are similar determinants of oral diseases and non-communicable diseases, it is necessary to combine policies on accessible dental services with reduction of sugar and tobacco consumption, increasing health literacy, reducing inequalities and access barriers. In this case, the important thing is not only how much expenditure is described as preventive, but also what type of intervention is financed and to whom it is provided.

The PES may be useful for descriptive comparisons of the preventive share within selected functions, but it cannot by itself assess whether preventive resources are effectively translated into population health gains. Future monitoring frameworks should trace the conversion of financial inputs into disease-specific services, implementation, effective coverage, quality, equity, utilization, and measurable population benefit.

### 4.4. Strengths and Limitations

This study used harmonized System of Health Accounts data and examined up to 27 EU Member States over eight years. The longitudinal ecological design is estimated with-in-country associations while controlling for country and year effects. Outcomes were GBD modeled prevalence estimates; the caries series used an all-ages setting and the periodontal series an age-standardized setting, so the two outcomes do not share an identical age basis. Age structure was not included as a separate time-varying covariate. Changes in population age composition may therefore contribute to within-country variation in all-ages caries prevalence, whereas the periodontal outcome was age-standardized. Country fixed effects account for stable demographic differences but do not remove confounding arising from demographic changes during follow-up. Absolute expenditure, relative allocation, and functional shares were examined for both outcomes. Interpretation is limited by the observational design: country-level associations cannot be extrapolated to individuals, and fixed effects do not remove reverse causality or unmeasured time-varying confounding.

Second, HC.6 and HC.1 cover healthcare functions across disease areas and do not identify oral-health-specific spending or changes in dental service delivery. Cross-country harmonization under SHA 2011 supports comparability but cannot ensure that national reporting practices or service content are identical. The estimates therefore test associations between system-wide expenditure categories and modeled oral-health outcomes, not the effects of oral-health spending.

Third, the panel includes only eight years and limited within-country change in the prevalence estimates. GBD outcomes are modeled estimates rather than annual clinical examinations; temporal smoothing, changing data inputs, and uncertainty in the estimates may affect apparent year-to-year variation. Mean annual cross-country caries prevalence declined from 35.01% in 2016 to 34.42% in 2023 (−0.59 percentage points), while periodontal prevalence changed from 11.17% to 11.07% (−0.10 percentage points). Across the 27 countries, the mean absolute annual within-country change was 0.153 percentage points for caries and 0.064 for periodontal disease, compared with mean GBD 95% uncertainty-interval widths of 10.98 and 5.31 percentage points, respectively. The 2016–2023 country-specific changes ranged from −2.03 to +0.17 percentage points for caries and from −2.45 to +0.29 percentage points for periodontal disease. These intervals are much wider than the typical annual movements in the modeled point estimates. GBD uncertainty limits were used in an exploratory weighting sensitivity analysis, but model uncertainty was not fully propagated through the regression analyses; the primary model confidence intervals therefore understate total uncertainty. The one-year lag is a feasible short-panel sensitivity analysis, not a biologically sufficient latency for cumulative caries or periodontal disease. Evidence on caries progression in permanent dentition among children and adolescents and on cumulative periodontal attachment loss over decades in adult men supports the plausibility of longer disease trajectories but does not establish the appropriate latency for these EU estimates [34,35]. The suggestion that effects may take longer was not tested here. Excluding 2020–2021 did not materially alter the direction of the reported associations.

Fourth, important time-varying determinants—including dental insurance, out-of-pocket costs, service utilization, fluoride exposure, diet, smoking, inequality, governance, and national oral-health policies—could not be incorporated consistently. Residual confounding remains possible, and fixed effects do not remove reverse causality: higher disease burden may prompt greater preventive or curative spending. Dentist density does not capture geographic distribution, affordability, coverage, workforce activity, or utilization; six country-year observations were unavailable in the HC.1 work-force-adjusted models, all from Luxembourg (2018–2023). The primary HC.1 analysis excluded Ireland, Italy, and Sweden because exact HC.1 expenditure was unavailable for all eight years. Their country-period means differed descriptively from those of included countries: caries prevalence was 33.76% versus 34.93%, periodontal prevalence 9.77% versus 11.38%, GDP per capita 45,155 versus 31,592 PPS, preventive expenditure 133.60 versus 92.48 PPS per inhabitant, HC.1 + HC.2 expenditure 1851.06 versus 1475.69 PPS per inhabitant, and dentist density 72.83 versus 82.17 per 100,000 inhabitants (means of country-specific period means). These comparisons use only three excluded countries and are descriptive, not inferential; the two analytic panels may therefore represent different populations (Table S4). Finally, PES and expenditure ratios cannot identify program content, implementation, or effective coverage.

Despite these limitations, the study provides a country-level assessment of whether broad expenditure composition is associated with modeled population oral health. Future research should link disease-specific expenditure to program content, populations served, implementation, effective coverage, quality, equity, and clinical or surveillance outcomes over longer periods, while propagating outcome uncertainty and addressing time-varying confounding.

Detailed within-country studies could complement cross-country panels by linking changes in oral-health financing and benefit packages to specific programs, service uptake, and subsequent outcomes. Such analyses should account for national reporting practices and examine whether preventive services reach populations with the greatest unmet need.

## 5. Conclusions

In this ecological panel of EU Member States, the primary analyses did not show that higher preventive expenditure or a greater preventive share within broad system-wide expenditure categories was associated with lower modeled oral-disease prevalence. A secondary log-ratio specification showed a positive association with caries, whereas the PES and untransformed ratio estimates did not; this functional-form sensitivity does not establish a general association or effect of preventive allocation. The positive adjusted association between preventive expenditure and caries was not reproduced in first differences. General curative expenditure was positively associated with modeled periodontal prevalence in several, but not all, specifications; need-driven allocation is one possible hypothesis, but this study cannot distinguish it from reverse causality, confounding, or other mechanisms. None of these ecological associations establishes causal effects or identifies oral-health-specific expenditure.

The findings show that broad expenditure categories alone cannot establish whether effective oral-health services reached populations or whether resources were used efficiently. The PES is a descriptive summary of expenditure composition, not a measure of program performance. Future evaluations should link oral-health-specific financing to service availability, implementation, effective coverage, utilization, quality, equity, and outcomes. Longer panels with detailed program and service data are needed to assess whether specific investments are associated with subsequent changes in population oral health.

## Figures and Tables

**Table 1:** Descriptive characteristics of the study variables, 2016–2023.

Variable	N	Mean	SD	Median	Q1–Q3	Minimum	Maximum
Caries prevalence (%)	216	34.80	6.00	36.74	31.20–39.55	22.29	44.58
Periodontal disease prevalence (%)	216	11.20	4.02	10.55	9.50–13.34	2.40	22.97
Preventive expenditure (PPS per inhabitant)	216	97.05	80.64	76.79	42.49–123.97	10.76	499.74
Curative expenditure (PPS per inhabitant)	192	1373.30	525.26	1288.84	942.39–1809.97	418.87	2625.14
Curative and rehabilitative expenditure (PPS per inhabitant)	216	1517.40	579.94	1435.00	1021.17–1977.28	444.54	2962.99
Preventive Expenditure Share, HC.1 (%)	192	5.74	3.04	5.19	3.74–6.75	1.47	18.46
Preventive Expenditure Share, HC.1 + HC.2 (%)	216	5.56	2.80	5.07	3.58–6.56	1.41	17.14
Preventive share of CHE (%)	216	3.28	1.70	3.07	2.11–3.97	0.77	10.44
Curative share of CHE (%)	192	52.34	4.83	52.61	48.93–56.06	41.77	61.28
GDP per capita (PPS)	216	33,098.69	14,419.99	29,701.35	23,918.53–37,389.20	14,411.00	95,129.10
Dentists per 100,000 inhabitants	209	80.83	21.61	76.33	67.45–95.56	34.04	133.71

**Table 2:** Two-way fixed effects estimate for preventive and curative health expenditure in relation to oral health outcomes.

Variable	Model 1, β (95% CI)	*p*	Model 2, β (95% CI)	*p*	Model 3, β (95% CI)	*p*
Panel A. Caries prevalence						
ln(Preventive expenditure)	0.299 (−0.015, 0.613)	0.061	0.291 (0.028, 0.554)	0.032	0.237 (0.044, 0.431)	0.018
ln(Curative expenditure)	−0.742 (−1.453, −0.030)	0.042	−0.107 (−0.798, 0.584)	0.751	−0.125 (−0.806, 0.556)	0.708
ln(GDP per capita)	—	—	−2.027 (−3.656, −0.398)	0.017	−1.676 (−3.358, 0.007)	0.051
Dentists per 100,000 inhabitants	—	—	—	—	−0.0068 (−0.0146, 0.0011)	0.088
Observations	192		192		186	
Countries	24		24		24	
Partial *R*^2^	0.131		0.200		0.236	
Panel B. Periodontal disease prevalence						
ln(Preventive expenditure)	0.079 (−0.179, 0.337)	0.532	0.078 (−0.172, 0.329)	0.523	0.014 (−0.107, 0.135)	0.817
ln(Curative expenditure)	0.370 (0.167, 0.572)	0.001	0.416 (0.038, 0.794)	0.032	0.388 (0.027, 0.749)	0.036
ln(GDP per capita)	—	—	−0.148 (−1.346, 1.049)	0.800	0.378 (−0.265, 1.022)	0.236
Dentists per 100,000 inhabitants	—	—	—	—	−0.0075 (−0.0128, −0.0022)	0.008
Observations	192		192		186	
Countries	24		24		24	
Partial *R*^2^	0.137		0.140		0.406	

Note: The dependent variables were expressed in percentage points. Preventive expenditure, curative expenditure, and GDP per capita were natural-log transformed. Model 1 included preventive and curative expenditure. Model 2 additionally included GDP per capita. Model 3 additionally included dental workforce density. All models included country and year fixed effects. Standard errors were clustered at the country level. Partial R^2^ represents the incremental explanatory contribution of the time-varying covariates beyond country and year fixed effects. Abbreviations: CI, confidence interval; GDP, gross domestic product.

**Table 3:** Associations of preventive spending orientation and functional expenditure share with oral health outcomes.

Variable	Relative Allocation, HC.1: β (95% CI)	*p*	Functional Shares: β (95% CI)	*p*	Relative Allocation, HC.1 + HC.2: β (95% CI)	*p*
Panel A. Caries prevalence						
Preventive Expenditure Share	0.023 (−0.013, 0.058)	0.196	—	—	0.024 (−0.012, 0.061)	0.182
P/C ratio	1.570 (−1.136, 4.275)	0.242	—	—	1.757 (−1.115, 4.628)	0.220
ln(P/C ratio)	0.281 (0.026, 0.537)	0.032	—	—	0.274 (0.022, 0.527)	0.034
Preventive share of CHE	—	—	0.042 (−0.036, 0.120)	0.280	—	—
Curative share of CHE	—	—	0.010 (−0.028, 0.047)	0.596	—	—
ln(GDP per capita)	−1.961 (−3.337, −0.584)	0.007	−2.123 (−3.659, −0.588)	0.009	−1.605 (−2.763, −0.447)	0.008
Observations	192		192		216	
Countries	24		24		27	
Partial *R*^2^	0.148		0.147		0.123	
Panel B. Periodontal disease prevalence						
Preventive Expenditure Share	0.005 (−0.020, 0.031)	0.666	—	—	0.035 (−0.028, 0.097)	0.263
P/C ratio	0.383 (−1.517, 2.283)	0.680	—	—	2.705 (−2.218, 7.629)	0.269
ln(P/C ratio)	0.052 (−0.190, 0.294)	0.662	—	—	0.264 (−0.194, 0.722)	0.246
Preventive share of CHE	—	—	0.027 (−0.033, 0.086)	0.368	—	—
Curative share of CHE	—	—	0.020 (−0.008, 0.048)	0.159	—	—
ln(GDP per capita)	0.419 (−0.383, 1.222)	0.291	0.193 (−0.847, 1.233)	0.705	1.688 (−0.700, 4.076)	0.158
Observations	192		192		216	
Countries	24		24		27	
Partial *R*^2^	0.033		0.123		0.098	

Note: PES, HC.1 represents preventive expenditure divided by the sum of preventive and curative expenditure. PES, HC.1 + HC.2 replaces exact curative expenditure with combined curative and rehabilitative-care expenditure. P/C represents preventive expenditure divided by curative expenditure; P/(C + R) uses combined curative and rehabilitative-care expenditure in the denominator. The functional-share specification included preventive and curative expenditure as percentages of total current healthcare expenditure. All models included GDP per capita, country fixed effects, and year fixed effects. Standard errors were clustered at the country level. Abbreviations: CHE, current health expenditure; CI, confidence interval; GDP, gross domestic product; HC.1, curative care; HC.2, rehabilitative care; PES, Preventive Expenditure Share; P/C, preventive-to-curative expenditure ratio; P/(C + R), preventive-to-curative-and-rehabilitative expenditure ratio.

## Data Availability

The data presented in this study are openly available in Global Burden of Disease 2023 at https://doi.org/10.1016/S0140-6736(25)01637-X [32].

## Supplementary Materials

The following supporting information can be downloaded at: https://www.mdpi.com/article/10.3390/healthcare14213659/s1, Table S1: Pearson correlation matrix for the study variables; Table S2: Diagnostic assessment of the primary and complete-panel fixed-effects models; Table S3: Coefficients from the primary and sensitivity analyses; Table S4: Country-year availability of exact HC.1 expenditure and dental workforce data; Figure S1: Partial relationships between caries prevalence and relative-allocation measures.
