# Supplementary material for: Health Financing and Functional Health Expenditure Allocation in Relation to Oral Health: A Longitudinal Ecological Panel Study of EU Member States Between 2016 and 2023
Source: Healthcare (Basel). 2026 Oct 28;14(21):3659. doi: 10.3390/healthcare14213659 (PMC13763109; doi:10.3390/healthcare14213659)
Supplement: Supplementary file 1 [file healthcare-14-03659-s001.zip › healthcare-4594963-supplementary.pdf]

Table S1. Pearson correlation matrix for the study variables.

Variable	1	2	3	4	5	6	7	8	9	10	11
1. Caries prevalence	1										
2. Periodontal disease prevalence	-0.506	1									
3. Preventive expenditure	-0.386	0.196	1								
4. Curative expenditure	-0.490	0.358	0.679	1							
5. Curative and rehabilitative expenditure	-0.448	0.287	0.667	0.990	1						
6. PES, HC.1	-0.291	0.113	0.893	0.380	0.368	1					
7. PES, HC.1 + HC.2	-0.303	0.090	0.875	0.355	0.325	0.997	1				
8. Preventive share of CHE	-0.310	0.092	0.863	0.359	0.322	0.985	0.989	1			
9. Curative share of CHE	-0.001	-0.032	-0.299	-0.156	-0.226	-0.289	-0.250	-0.142	1		
10. GDP per capita	-0.215	0.066	0.472	0.706	0.720	0.284	0.234	0.225	-0.277	1	
11. Dentists per 100,000 inhabitants	0.162	-0.007	-0.214	-0.263	-0.315	-0.088	-0.074	-0.054	0.418	-0.349	1

Note: Correlations were calculated using pairwise complete observations. The number of observations therefore varied according to data availability. Oral health outcomes, preventive expenditure, combined curative and rehabilitative expenditure, PES, HC.1 + HC.2, preventive share of current health expenditure, and GDP per capita were available for 216 observations. Exact curative expenditure, PES, HC.1, and curative share of current health expenditure were available for 192 observations. Dental workforce density was available for 209 observations. Correlations are pooled and unadjusted for country and year effects; they are presented for descriptive purposes only. Abbreviations: CHE, current health expenditure; GDP, gross domestic product; HC.1, curative care; HC.2, rehabilitative care.

Table S2. Diagnostic assessment of the primary and complete-panel fixed-effects models.

Model	Residual AR(1) coefficient	AR(1) test *p*	Brown–Forsythe statistic	Brown–Forsythe *p*	Pesaran CD statistic	Pesaran CD *p*
Caries, primary HC.1 model	0.460	<0.001	2.449	<0.001	-0.795	0.426
Periodontal disease, primary HC.1 model	0.670	<0.001	17.268	<0.001	-0.235	0.814
Caries, HC.1 + HC.2 model	0.477	<0.001	2.455	<0.001	-0.757	0.449
Periodontal disease, HC.1 + HC.2 model	0.690	<0.001	3.937	<0.001	4.960	<0.001

Multicollinearity assessment

Explanatory variable	VIF
ln(Preventive expenditure)	2.41
ln(Curative expenditure)	4.45
ln(GDP per capita)	3.24

Note: The residual AR(1) test evaluated first-order correlation in model residuals. The Brown–Forsythe test evaluated differences in residual variance across countries. The Pesaran CD test evaluated cross-sectional dependence. VIF values were calculated for the time-varying explanatory variables included in the primary GDP-adjusted model.

Abbreviations: AR(1), first-order autoregressive process; GDP, gross domestic product; HC.1, curative care; HC.2, rehabilitative care; VIF, variance inflation factor.

Coefficients are expressed as percentage-point differences in the outcome per one-unit change in the listed exposure (log expenditure where applicable). All models include country and year effects when specified by the model name; GDP-adjusted models include log GDP per capita. For first-difference models, coefficients refer to changes in the transformed variables. The leave-one-country-out confidence intervals are reconstructed from the reported country-clustered standard errors using a t critical value with 22 degrees of freedom (23 retained country clusters); corresponding P values are those reported in the supplied model output. Driscoll–Kraay inference is available in the original coefficient file and should be considered cautiously because T=8. Exploratory inverse-variance-weighted estimates use weights $1/SE^2$, with SE approximated as the GBD 95% uncertainty-interval width divided by 3.92; this approximation does not capture all uncertainty from the GBD modelling process. Abbreviations: FE, fixed effects; GDP, gross domestic product; HC.1, curative care; HC.2, rehabilitative care; OLS, ordinary least squares.

Table S3. Coefficients from the primary and sensitivity analyses.

Outcome	Sensitivity specification	Exposure	β (95% CI)	P	N	Countries	Years
Caries prevalence	Primary FE, Model 1 (HC.1)	Preventive expenditure	0.299 (-0.015, 0.613)	0.061	192	24	8
Caries prevalence	Primary FE, Model 1 (HC.1)	Curative expenditure	-0.742 (-1.453, -0.030)	0.042	192	24	8
Caries prevalence	Primary FE, Model 2 (GDP-adjusted)	Preventive expenditure	0.291 (0.028, 0.554)	0.032	192	24	8
Caries prevalence	Primary FE, Model 2 (GDP-adjusted)	Curative expenditure	-0.107 (-0.798, 0.584)	0.751	192	24	8
Caries prevalence	Workforce-adjusted FE	Preventive expenditure	0.237 (0.044, 0.431)	0.018	186	24	8
Caries prevalence	Workforce-adjusted FE	Curative expenditure	-0.125 (-0.806, 0.556)	0.708	186	24	8
Caries prevalence	One-year lagged FE	Preventive expenditure	0.282 (0.026, 0.538)	0.032	168	24	7
Caries prevalence	One-year lagged FE	Curative expenditure	-0.028 (-0.731, 0.675)	0.935	168	24	7
Caries prevalence	First-difference model	Preventive expenditure	0.033 (-0.103, 0.170)	0.617	168	24	7
Caries prevalence	First-difference model	Curative expenditure	0.074 (-0.372, 0.520)	0.734	168	24	7
Caries prevalence	Excluding 2020–2021	Preventive expenditure	0.410 (0.108, 0.711)	0.010	144	24	6
Caries prevalence	Excluding 2020–2021	Curative expenditure	-0.368 (-1.796, 1.061)	0.600	144	24	6
Caries prevalence	Alternative treatment measure (HC.1+HC.2)	Preventive expenditure	0.276 (0.018, 0.533)	0.037	216	27	8
Caries prevalence	Alternative treatment measure (HC.1+HC.2)	Curative + rehabilitative expenditure	-0.238 (-0.921, 0.445)	0.481	216	27	8

Outcome	Sensitivity specification	Exposure	β (95% CI)	P	N	Countries	Years
Caries prevalence	Random-effects estimator	Preventive expenditure	0.284 (0.036, 0.531)	0.026	192	24	8
Caries prevalence	Random-effects estimator	Curative expenditure	-0.117 (-0.768, 0.534)	0.713	192	24	8
Caries prevalence	Pooled OLS estimator	Preventive expenditure	-3.069 (-7.520, 1.382)	0.167	192	24	8
Caries prevalence	Pooled OLS estimator	Curative expenditure	-4.595 (-17.143, 7.954)	0.456	192	24	8
Caries prevalence	Balanced workforce sensitivity (excludes Luxembourg)	Preventive expenditure	0.238 (0.044, 0.432)	0.019	184	23	8
Caries prevalence	Balanced workforce sensitivity (excludes Luxembourg)	Curative expenditure	-0.121 (-0.805, 0.562)	0.716	184	23	8
Periodontal prevalence	Primary FE, Model 1 (HC.1)	Preventive expenditure	0.079 (-0.179, 0.337)	0.532	192	24	8
Periodontal prevalence	Primary FE, Model 1 (HC.1)	Curative expenditure	0.370 (0.167, 0.572)	0.001	192	24	8
Periodontal prevalence	Primary FE, Model 2 (GDP-adjusted)	Preventive expenditure	0.078 (-0.172, 0.329)	0.523	192	24	8
Periodontal prevalence	Primary FE, Model 2 (GDP-adjusted)	Curative expenditure	0.416 (0.038, 0.794)	0.032	192	24	8
Periodontal prevalence	Workforce-adjusted FE	Preventive expenditure	0.014 (-0.107, 0.135)	0.817	186	24	8
Periodontal prevalence	Workforce-adjusted FE	Curative expenditure	0.388 (0.027, 0.749)	0.036	186	24	8
Periodontal prevalence	One-year lagged FE	Preventive expenditure	0.062 (-0.166, 0.289)	0.581	168	24	7
Periodontal prevalence	One-year lagged FE	Curative expenditure	0.418 (-0.021, 0.858)	0.061	168	24	7
Periodontal prevalence	First-difference model	Preventive expenditure	0.028 (-0.049, 0.104)	0.463	168	24	7
Periodontal prevalence	First-difference model	Curative expenditure	0.262 (0.034, 0.490)	0.026	168	24	7
Periodontal prevalence	Excluding 2020–2021	Preventive expenditure	0.066 (-0.236, 0.367)	0.656	144	24	6
Periodontal prevalence	Excluding 2020–2021	Curative expenditure	0.332 (0.005, 0.659)	0.047	144	24	6
Periodontal prevalence	Alternative treatment measure (HC.1+HC.2)	Preventive expenditure	0.296 (-0.175, 0.766)	0.207	216	27	8

Outcome	Sensitivity specification	Exposure	β (95% CI)	P	N	Countries	Years
Periodontal prevalence	Alternative treatment measure (HC.1+HC.2)	Curative + rehabilitative expenditure	0.564 (0.006, 1.123)	0.048	216	27	8
Periodontal prevalence	Random-effects estimator	Preventive expenditure	0.079 (-0.155, 0.313)	0.491	192	24	8
Periodontal prevalence	Random-effects estimator	Curative expenditure	0.416 (0.063, 0.769)	0.023	192	24	8
Periodontal prevalence	Pooled OLS estimator	Preventive expenditure	0.349 (-2.070, 2.769)	0.768	192	24	8
Periodontal prevalence	Pooled OLS estimator	Curative expenditure	2.535 (-6.302, 11.373)	0.559	192	24	8
Periodontal prevalence	Balanced workforce sensitivity (excludes Luxembourg)	Preventive expenditure	0.014 (-0.108, 0.135)	0.819	184	23	8
Periodontal prevalence	Balanced workforce sensitivity (excludes Luxembourg)	Curative expenditure	0.387 (0.025, 0.749)	0.037	184	23	8
Caries prevalence	Leave-one-country-out (omit AT)	Preventive expenditure	0.341 (0.077, 0.606)	0.014	184	23	8
Caries prevalence	Leave-one-country-out (omit AT)	Curative expenditure	-0.148 (-0.838, 0.543)	0.662	184	23	8
Caries prevalence	Leave-one-country-out (omit BE)	Preventive expenditure	0.305 (0.057, 0.552)	0.018	184	23	8
Caries prevalence	Leave-one-country-out (omit BE)	Curative expenditure	-0.021 (-0.674, 0.633)	0.949	184	23	8
Caries prevalence	Leave-one-country-out (omit BG)	Preventive expenditure	0.297 (0.027, 0.566)	0.033	184	23	8
Caries prevalence	Leave-one-country-out (omit BG)	Curative expenditure	-0.082 (-0.784, 0.619)	0.810	184	23	8
Caries prevalence	Leave-one-country-out (omit CY)	Preventive expenditure	0.302 (0.034, 0.570)	0.029	184	23	8
Caries prevalence	Leave-one-country-out (omit CY)	Curative expenditure	-0.014 (-0.714, 0.686)	0.968	184	23	8
Caries prevalence	Leave-one-country-out (omit CZ)	Preventive expenditure	0.288 (0.013, 0.562)	0.041	184	23	8
Caries prevalence	Leave-one-country-out (omit CZ)	Curative expenditure	-0.007 (-0.640, 0.627)	0.983	184	23	8
Caries prevalence	Leave-one-country-out (omit DE)	Preventive expenditure	0.306 (0.041, 0.571)	0.025	184	23	8
Caries prevalence	Leave-one-country-out (omit DE)	Curative expenditure	-0.107 (-0.799, 0.584)	0.750	184	23	8
Caries prevalence	Leave-one-country-out (omit DK)	Preventive expenditure	0.308 (0.035, 0.581)	0.029	184	23	8
Caries prevalence	Leave-one-country-out (omit DK)	Curative expenditure	-0.127 (-0.872, 0.617)	0.726	184	23	8

Outcome	Sensitivity specification	Exposure	β (95% CI)	P	N	Countries	Years
Caries prevalence	Leave-one-country-out (omit EE)	Preventive expenditure	0.285 (0.022, 0.549)	0.035	184	23	8
Caries prevalence	Leave-one-country-out (omit EE)	Curative expenditure	-0.081 (-0.788, 0.626)	0.815	184	23	8
Caries prevalence	Leave-one-country-out (omit EL)	Preventive expenditure	0.296 (0.015, 0.578)	0.040	184	23	8
Caries prevalence	Leave-one-country-out (omit EL)	Curative expenditure	-0.107 (-0.803, 0.589)	0.753	184	23	8
Caries prevalence	Leave-one-country-out (omit ES)	Preventive expenditure	0.291 (0.022, 0.560)	0.035	184	23	8
Caries prevalence	Leave-one-country-out (omit ES)	Curative expenditure	-0.089 (-0.794, 0.615)	0.795	184	23	8
Caries prevalence	Leave-one-country-out (omit FI)	Preventive expenditure	0.282 (0.010, 0.553)	0.043	184	23	8
Caries prevalence	Leave-one-country-out (omit FI)	Curative expenditure	-0.138 (-0.869, 0.592)	0.698	184	23	8
Caries prevalence	Leave-one-country-out (omit FR)	Preventive expenditure	0.292 (0.023, 0.562)	0.035	184	23	8
Caries prevalence	Leave-one-country-out (omit FR)	Curative expenditure	-0.114 (-0.813, 0.586)	0.739	184	23	8
Caries prevalence	Leave-one-country-out (omit HR)	Preventive expenditure	0.280 (-0.004, 0.564)	0.053	184	23	8
Caries prevalence	Leave-one-country-out (omit HR)	Curative expenditure	-0.098 (-0.780, 0.584)	0.769	184	23	8
Caries prevalence	Leave-one-country-out (omit HU)	Preventive expenditure	0.309 (0.033, 0.585)	0.030	184	23	8
Caries prevalence	Leave-one-country-out (omit HU)	Curative expenditure	-0.092 (-0.782, 0.599)	0.786	184	23	8
Caries prevalence	Leave-one-country-out (omit LT)	Preventive expenditure	0.277 (0.023, 0.532)	0.034	184	23	8
Caries prevalence	Leave-one-country-out (omit LT)	Curative expenditure	-0.105 (-0.840, 0.630)	0.771	184	23	8
Caries prevalence	Leave-one-country-out (omit LU)	Preventive expenditure	0.294 (0.025, 0.563)	0.034	184	23	8
Caries prevalence	Leave-one-country-out (omit LU)	Curative expenditure	-0.082 (-0.840, 0.676)	0.824	184	23	8
Caries prevalence	Leave-one-country-out (omit LV)	Preventive expenditure	0.302 (0.032, 0.572)	0.030	184	23	8
Caries prevalence	Leave-one-country-out (omit LV)	Curative expenditure	-0.405 (-1.106, 0.295)	0.243	184	23	8
Caries prevalence	Leave-one-country-out (omit MT)	Preventive expenditure	0.265 (-0.051, 0.582)	0.096	184	23	8
Caries prevalence	Leave-one-country-out (omit MT)	Curative expenditure	-0.117 (-0.829, 0.595)	0.737	184	23	8
Caries prevalence	Leave-one-country-out (omit NL)	Preventive expenditure	0.279 (0.023, 0.535)	0.034	184	23	8
Caries prevalence	Leave-one-country-out (omit NL)	Curative expenditure	-0.004 (-0.648, 0.639)	0.989	184	23	8

Outcome	Sensitivity specification	Exposure	β (95% CI)	P	N	Countries	Years
Caries prevalence	Leave-one-country-out (omit PL)	Preventive expenditure	0.168 (-0.019, 0.355)	0.076	184	23	8
Caries prevalence	Leave-one-country-out (omit PL)	Curative expenditure	-0.239 (-0.974, 0.497)	0.508	184	23	8
Caries prevalence	Leave-one-country-out (omit PT)	Preventive expenditure	0.312 (0.059, 0.566)	0.018	184	23	8
Caries prevalence	Leave-one-country-out (omit PT)	Curative expenditure	-0.189 (-0.905, 0.526)	0.589	184	23	8
Caries prevalence	Leave-one-country-out (omit RO)	Preventive expenditure	0.292 (0.022, 0.563)	0.036	184	23	8
Caries prevalence	Leave-one-country-out (omit RO)	Curative expenditure	-0.155 (-0.946, 0.637)	0.689	184	23	8
Caries prevalence	Leave-one-country-out (omit SI)	Preventive expenditure	0.295 (0.028, 0.561)	0.032	184	23	8
Caries prevalence	Leave-one-country-out (omit SI)	Curative expenditure	-0.101 (-0.791, 0.588)	0.763	184	23	8
Caries prevalence	Leave-one-country-out (omit SK)	Preventive expenditure	0.299 (0.001, 0.597)	0.049	184	23	8
Caries prevalence	Leave-one-country-out (omit SK)	Curative expenditure	-0.078 (-0.770, 0.615)	0.819	184	23	8
Periodontal prevalence	Leave-one-country-out (omit AT)	Preventive expenditure	0.091 (-0.187, 0.369)	0.503	184	23	8
Periodontal prevalence	Leave-one-country-out (omit AT)	Curative expenditure	0.409 (0.042, 0.777)	0.031	184	23	8
Periodontal prevalence	Leave-one-country-out (omit BE)	Preventive expenditure	0.078 (-0.177, 0.332)	0.534	184	23	8
Periodontal prevalence	Leave-one-country-out (omit BE)	Curative expenditure	0.402 (0.015, 0.789)	0.042	184	23	8
Periodontal prevalence	Leave-one-country-out (omit BG)	Preventive expenditure	0.084 (-0.173, 0.342)	0.503	184	23	8
Periodontal prevalence	Leave-one-country-out (omit BG)	Curative expenditure	0.437 (0.053, 0.822)	0.028	184	23	8
Periodontal prevalence	Leave-one-country-out (omit CY)	Preventive expenditure	0.087 (-0.168, 0.341)	0.488	184	23	8
Periodontal prevalence	Leave-one-country-out (omit CY)	Curative expenditure	0.514 (0.112, 0.915)	0.015	184	23	8
Periodontal prevalence	Leave-one-country-out (omit CZ)	Preventive expenditure	0.083 (-0.174, 0.340)	0.508	184	23	8
Periodontal prevalence	Leave-one-country-out (omit CZ)	Curative expenditure	0.396 (0.024, 0.767)	0.038	184	23	8

Outcome	Sensitivity specification	Exposure	β (95% CI)	P	N	Countries	Years
Periodontal prevalence	Leave-one-country-out (omit DE)	Preventive expenditure	0.084 (-0.173, 0.342)	0.503	184	23	8
Periodontal prevalence	Leave-one-country-out (omit DE)	Curative expenditure	0.411 (0.037, 0.784)	0.033	184	23	8
Periodontal prevalence	Leave-one-country-out (omit DK)	Preventive expenditure	0.069 (-0.190, 0.327)	0.587	184	23	8
Periodontal prevalence	Leave-one-country-out (omit DK)	Curative expenditure	0.416 (-0.000, 0.832)	0.050	184	23	8
Periodontal prevalence	Leave-one-country-out (omit EE)	Preventive expenditure	0.080 (-0.170, 0.330)	0.515	184	23	8
Periodontal prevalence	Leave-one-country-out (omit EE)	Curative expenditure	0.429 (0.034, 0.825)	0.035	184	23	8
Periodontal prevalence	Leave-one-country-out (omit EL)	Preventive expenditure	0.101 (-0.159, 0.361)	0.431	184	23	8
Periodontal prevalence	Leave-one-country-out (omit EL)	Curative expenditure	0.384 (0.027, 0.741)	0.036	184	23	8
Periodontal prevalence	Leave-one-country-out (omit ES)	Preventive expenditure	0.085 (-0.171, 0.340)	0.498	184	23	8
Periodontal prevalence	Leave-one-country-out (omit ES)	Curative expenditure	0.405 (0.025, 0.786)	0.038	184	23	8
Periodontal prevalence	Leave-one-country-out (omit FI)	Preventive expenditure	0.080 (-0.171, 0.330)	0.517	184	23	8
Periodontal prevalence	Leave-one-country-out (omit FI)	Curative expenditure	0.447 (0.051, 0.843)	0.029	184	23	8
Periodontal prevalence	Leave-one-country-out (omit FR)	Preventive expenditure	0.079 (-0.175, 0.333)	0.525	184	23	8
Periodontal prevalence	Leave-one-country-out (omit FR)	Curative expenditure	0.411 (0.027, 0.795)	0.037	184	23	8
Periodontal prevalence	Leave-one-country-out (omit HR)	Preventive expenditure	0.082 (-0.170, 0.334)	0.508	184	23	8
Periodontal prevalence	Leave-one-country-out (omit HR)	Curative expenditure	0.407 (0.036, 0.778)	0.033	184	23	8
Periodontal prevalence	Leave-one-country-out (omit HU)	Preventive expenditure	0.089 (-0.173, 0.352)	0.487	184	23	8
Periodontal prevalence	Leave-one-country-out (omit HU)	Curative expenditure	0.418 (0.042, 0.794)	0.031	184	23	8

Outcome	Sensitivity specification	Exposure	β (95% CI)	P	N	Countries	Years
Periodontal prevalence	Leave-one-country-out (omit LT)	Preventive expenditure	0.074 (-0.175, 0.323)	0.543	184	23	8
Periodontal prevalence	Leave-one-country-out (omit LT)	Curative expenditure	0.417 (0.043, 0.792)	0.030	184	23	8
Periodontal prevalence	Leave-one-country-out (omit LU)	Preventive expenditure	0.076 (-0.180, 0.332)	0.543	184	23	8
Periodontal prevalence	Leave-one-country-out (omit LU)	Curative expenditure	0.431 (0.031, 0.831)	0.036	184	23	8
Periodontal prevalence	Leave-one-country-out (omit LV)	Preventive expenditure	0.082 (-0.173, 0.336)	0.514	184	23	8
Periodontal prevalence	Leave-one-country-out (omit LV)	Curative expenditure	0.482 (-0.002, 0.966)	0.051	184	23	8
Periodontal prevalence	Leave-one-country-out (omit MT)	Preventive expenditure	0.112 (-0.163, 0.387)	0.409	184	23	8
Periodontal prevalence	Leave-one-country-out (omit MT)	Curative expenditure	0.422 (0.042, 0.802)	0.031	184	23	8
Periodontal prevalence	Leave-one-country-out (omit NL)	Preventive expenditure	0.077 (-0.173, 0.328)	0.528	184	23	8
Periodontal prevalence	Leave-one-country-out (omit NL)	Curative expenditure	0.427 (0.034, 0.820)	0.034	184	23	8
Periodontal prevalence	Leave-one-country-out (omit PL)	Preventive expenditure	-0.055 (-0.120, 0.010)	0.095	184	23	8
Periodontal prevalence	Leave-one-country-out (omit PL)	Curative expenditure	0.228 (0.032, 0.423)	0.024	184	23	8
Periodontal prevalence	Leave-one-country-out (omit PT)	Preventive expenditure	0.080 (-0.175, 0.335)	0.522	184	23	8
Periodontal prevalence	Leave-one-country-out (omit PT)	Curative expenditure	0.420 (0.039, 0.801)	0.032	184	23	8
Periodontal prevalence	Leave-one-country-out (omit RO)	Preventive expenditure	0.083 (-0.172, 0.337)	0.507	184	23	8
Periodontal prevalence	Leave-one-country-out (omit RO)	Curative expenditure	0.407 (0.035, 0.779)	0.033	184	23	8
Periodontal prevalence	Leave-one-country-out (omit SI)	Preventive expenditure	0.081 (-0.172, 0.333)	0.515	184	23	8
Periodontal prevalence	Leave-one-country-out (omit SI)	Curative expenditure	0.418 (0.044, 0.792)	0.030	184	23	8

Outcome	Sensitivity specification	Exposure	β (95% CI)	P	N	Countries	Years
Periodontal prevalence	Leave-one-country-out (omit SK)	Preventive expenditure	0.084 (-0.202, 0.371)	0.548	184	23	8
Periodontal prevalence	Leave-one-country-out (omit SK)	Curative expenditure	0.414 (0.019, 0.810)	0.041	184	23	8
Caries	Weighted FE, GDP-adjusted	Preventive HC.6	0.356 (0.021, 0.691)	0.038	192	24	8
Caries	Weighted FE, GDP-adjusted	Curative HC.1	-0.109 (-0.768, 0.551)	0.736	192	24	8
Periodontal	Weighted FE, GDP-adjusted	Preventive HC.6	0.009 (-0.186, 0.203)	0.926	192	24	8
Periodontal	Weighted FE, GDP-adjusted	Curative HC.1	0.421 (0.050, 0.792)	0.028	192	24	8

Table S4. Country-year availability of exact HC.1 expenditure and dental workforce data.

Data item	Country	Unavailable years	Available observations
Exact curative expenditure (HC.1)	Ireland	2016–2023	0/8
Exact curative expenditure (HC.1)	Italy	2016–2023	0/8
Exact curative expenditure (HC.1)	Sweden	2016–2023	0/8
Dentist density	Luxembourg	2018–2023	2/8
Dentist density	Sweden	2023	7/8

Note: The HC.1 series contained Eurostat unavailable markers (":") in every study year for Ireland, Italy, and Sweden; those countries were excluded from the exact-HC.1 primary panel. Workforce observations were missing for Luxembourg in 2018–2023 and Sweden in 2023. The six Luxembourg observations account for the additional missing dentist-density values in the 24-country HC.1 workforce-adjusted models.

Figure S1. Partial relationships between caries prevalence and relative-allocation measures.

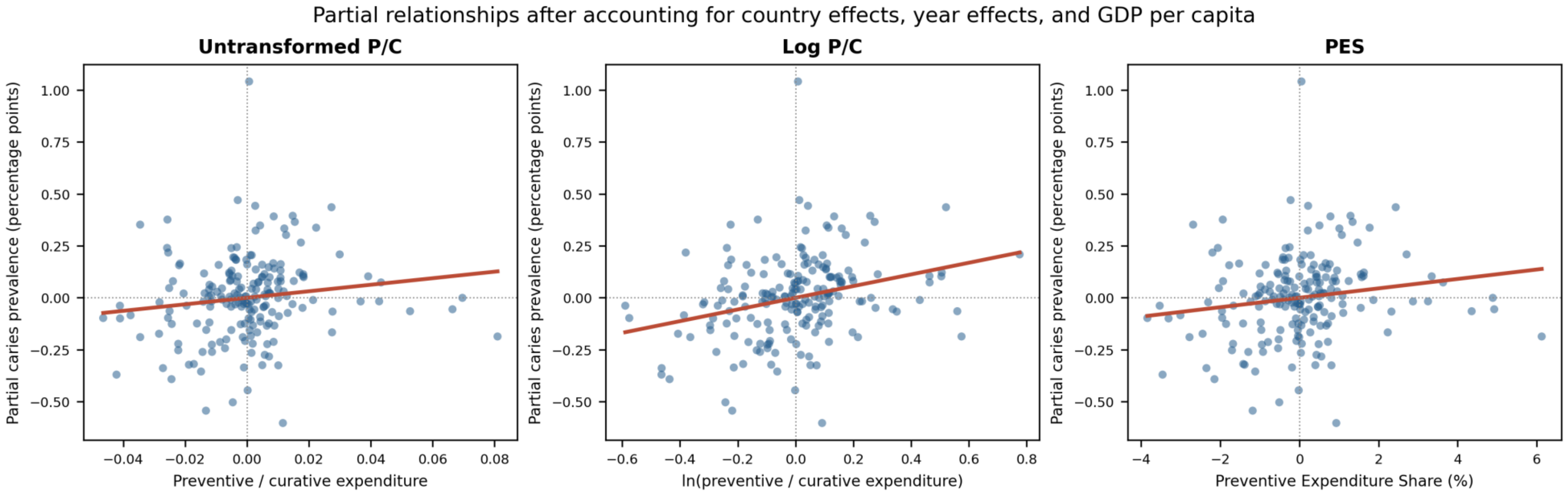

Note: Points are country-year observations from the 24-country HC.1 panel. The axes show partial residuals after accounting for country effects, year effects, and log GDP per capita; red lines are fitted linear relationships for each functional form. These plots are descriptive and do not establish causal effects.
